# “Since both of us are using antiretrovirals, we have been supportive to each other”: facilitators and barriers of pre-exposure prophylaxis use in heterosexual HIV serodiscordant couples in Kisumu, Kenya

**DOI:** 10.7448/IAS.19.1.21134

**Published:** 2016-12-12

**Authors:** Rena C Patel, Gaelen Stanford-Moore, Josephine Odoyo, Maria Pyra, Imeldah Wakhungu, Keerthana Anand, Elizabeth A Bukusi, Jared M Baeten, Joelle M Brown

**Affiliations:** 1 Division of Allergy and Infectious Diseases, Department of Medicine, University of Washington, Seattle, WA, USA; 2 School of Medicine, University of California, San Francisco, CA, USA; 3 Centre for Microbiology Research, Kenya Medical Research Institute, Kisumu, Kenya; 4 Department of Epidemiology, School of Public Health, University of Washington, Seattle, WA, USA; 5 Department of Preventive Medicine, Feinberg School of Medicine, Northwestern University, Chicago, IL, USA; 6 Department of Obstetrics and Gynecology, University of Washington, Seattle, WA, USA; 7 Departments of Epidemiology, Global Health, and Medicine, University of Washington, Seattle, WA, USA; 8 Departments of Epidemiology and Biostatistics, and Obstetrics, Gynecology, and Reproductive Sciences, University of California, San Francisco, CA, USA

**Keywords:** pre-exposure prophylaxis, barriers, facilitators, heterosexual, HIV serodiscordant couples, Kenya

## Abstract

**Introduction:**

Since 2015, the World Health Organization recommends pre-exposure prophylaxis (PrEP) for all persons at substantial risk for HIV, including HIV-uninfected partners in serodiscordant relationships in resource-limited settings. As PrEP moves from clinical trials to real-world use, understanding facilitators of and barriers to PrEP initiation and adherence is critical to successful PrEP implementation and rollout.

**Methods:**

We conducted 44 in-depth individual or couple interviews with 63 participants (30 without HIV and 33 with HIV) enrolled in the Partners Demonstration Project in Kisumu, Kenya, between August and September 2014. The semi-structured interviews discussed the following: 1) perceived advantages and disadvantages of antiretroviral therapy (ART)/PrEP; 2) reasons for accepting or declining ART/PrEP and 3) influence of prevention of transmission to partner or infant on ART/PrEP use. Transcripts from the interviews were iteratively analyzed using inductive content analysis.

**Results:**

Our study identified three key factors that may facilitate initiation of PrEP in this population. First, participants using PrEP felt reduced stress and increased trust in their HIV serodiscordant relationships. Second, greater community-wide knowledge of PrEP was thought to likely increase PrEP acceptance. Third, greater education and counselling by providers on PrEP use was also considered to likely increase the adoption of PrEP. We also identified three key barriers to initiation of and adherence to PrEP. First, most participants who declined PrEP expressed doubts about the relative additional effectiveness of PrEP in combination with other prevention tools. Second, perceived stigma related to PrEP use was an important barrier to PrEP initiation. Third, many struggled with overcoming perceived side effects or logistical challenges of taking daily PrEP, particularly when they themselves were not ill.

**Conclusions:**

Leveraging the facilitators and overcoming barriers to PrEP uptake may enhance the successful rollout of PrEP among HIV serodiscordant couples in Kenya and other areas in sub-Saharan Africa, thereby reducing sexual transmission of HIV. Further research focused on how best to provide counselling on combination HIV prevention tools in the context of PrEP use is a crucial next step to delivering PrEP.

## Introduction

Serodiscordant couples are an important target population for HIV prevention. In high-prevalence areas, 20 to 50% of HIV-infected individuals are in stable relationships with HIV-uninfected partners [1]. Depending on the population, serodiscordant couples can account for a substantial proportion of new HIV infections, from 13 to 55% [2,3]. Pre-exposure prophylaxis (PrEP) in clinical trials and open-label demonstrations is estimated to provide greater than 90% efficacy among those adherent in preventing HIV acquisition [4–6].

The World Health Organization now recommends PrEP for all persons at substantial risk for HIV, including HIV-uninfected partners in serodiscordant relationships in resource-limited settings [7]. As PrEP moves from clinical trials to real-world use, understanding barriers to initiation of and adherence to PrEP is critical to successful PrEP implementation. In studies to date, which are mostly conducted among populations of men who have sex with men, certain facilitators of and barriers to PrEP use have been identified [8–12]. Factors that appear to facilitate PrEP use and adherence include certain drug characteristics, such as daily over intermittent dosing and ability to use PrEP covertly or when condom use may be difficult, and perceived support from study staff, family and friends. Barriers include drug-related issues, such as side effects and pill characteristics; logistical issues around drug use, such as timely refills and travel; and social stigma, related to being mistakenly identified as HIV-infected and disclosure of use with sexual partners. Furthermore, clinical trials focused on HIV prevention among women have found that experiences with clinic personnel, mistrust of research, and the influence of male partners can either support or deter study participation and PrEP use [13–17]. However, it remains unclear if heterosexual couples participating in an open-label implementation project in resource-limited settings identify similar themes.

The current study was conducted within the Partners Demonstration Project, where the HIV-uninfected partners are offered PrEP as a “bridge” to antiretroviral therapy (ART) initiation and virologic suppression in the HIV-infected partners. In the Partners Demonstration Project, approximately 5% of the uninfected partners declined PrEP at enrolment [18]. We conducted a qualitative study with participants already enrolled in the Partners Demonstration Project in Kisumu, Kenya, to identify facilitators of and barriers to PrEP initiation and adherence. We report on those findings and discuss their implications for delivering PrEP in the region.

## Methods

The study was conducted in Kisumu, Kenya, between August and September 2014 among participants enrolled in the Partners Demonstration Project in Kisumu. Kisumu County has one of the highest HIV prevalence estimates in Kenya at 19.3%, compared with 6.0% nationally [19]. The Partners Demonstration Project is an open-label study implemented at four sites in Kenya and Uganda among 1013 high-risk HIV serodiscordant couples [20]. HIV serodiscordant couples at high risk for HIV transmission were enrolled; HIV-infected partners could not be using ART at enrolment in order to be eligible for the study. In Kisumu, HIV-infected partners were then referred to local HIV facilities to initiate ART per country guidelines, while the uninfected partner was offered PrEP as a “bridge” until the infected partner became eligible for ART and had confirmed plasma viral suppression 6 months after ART initiation.

For this qualitative study, we selected a quasi-random subsample of the participants enrolled in the Partners Demonstration Project who fell into one of four categories: 1) HIV-infected individual eligible for ART who initiated ART, 2) HIV-infected individual eligible for ART who declined ART initiation, 3) HIV-uninfected individual eligible for PrEP who initiated PrEP and 4) HIV-uninfected individual eligible for PrEP who declined PrEP initiation. We assessed initiation of ART or PrEP by the third month of the study enrolment, anticipating that three months was sufficient time for individuals to undergo counselling and decide on initiation of ART or PrEP. At the time of determining eligibility for this qualitative study in June 2014, ART eligibility was recommended for individuals with CD4 cell counts ≤350 or >350 cells/µL with a WHO clinical disease Stage III or IV [21]. After generating lists of potential participants in each of the four above categories, we randomly selected 20 participants to sample for this qualitative study, with the goal of conducting at least 10 interviews in each category. From this random sample, we attempted to invite an equal number of male and female participants. However, some of the categories were highly skewed by gender, for example, individuals who initiated ART were largely women. Potential participants were contacted by phone and, if interested, were scheduled for an interview at the study facility. All participants were encouraged to come with their study partners for the interview although it was ultimately their choice whether they came individually or with their partner.

By the end of June 2014, a total of 251 couples were enrolled in the Partners Demonstration Project. Out of the 80 individuals invited to participate in this qualitative study, we conducted 44 in-depth interviews with a total of 63 participants (30 without HIV and 33 with HIV). Of the interviews, 19 were conducted with the couples together, and an additional four interviews were conducted with both partners but individually; the remaining 21 interviews were conducted with only one individual from a couple. The interviewers were trained to specifically elicit comments from both partners and reflections on each other's responses during couple interviews.

The interviews were conducted by trained interviewers in DhoLuo and digitally recorded. We developed a semi-structure interview guide roughly informed by the Health Belief Model (particularly “perceived benefits” and “perceived barriers”) [22] and the Theory of Planned Behavior (particularly “behavioral intention” and “subjective norms”) [23]. The interviewers used this guide to prompt discussions on the following themes: 1) perceptions of ART, including advantages and disadvantages of each; 2) reasons for accepting or declining ART initiation; and 3) influence of prevention of transmission to partner or infant influencing ART/PrEP use. The interviewers then transcribed the initial five interviews in DhoLuo and then translated these into English. Another member of the study staff verified the accuracy of the English translations against the audio file and DhoLuo transcripts. Then, the interviewers translated the interview audio files directly into English. Transcripts were imported into Nvivo Version 10.1 for coding [24]. Transcripts from the interviews were iteratively analyzed using inductive content analysis. An initial codebook was developed from the interview guide, which was further refined with discussion and consensus as the initial transcripts were coded. The first 10 transcripts were double-coded by at least two members of the study team, and differences in coding were resolved through discussion until consensus was reached. After all the data were coded, the investigators used an iterative process of reading transcripts, applying inductive codes, comparing and contrasting codes, and identifying convergent and divergent themes.

The study received approval from the Kenya Medical Research Institute and the University of California, San Francisco ethics review boards. All the participants provided written informed consent.

## Results

Of the 30 HIV-uninfected participants interviewed, all eligible for PrEP, 19 (63%) had initiated PrEP during the study, 7 (23%) were female, 29 (97%) were married and 26 (87%) were of Luo ethnicity. The median age was 34 years (IQR 28–38), total number of living children was 3 (0 to 5), and number of living children with their study partner was 0 (0 to 2). The participants had been cohabitating with their study partners for a median number of 2 years (0.42 to 7) and had known their serodiscordant status for a median of 1 month (1 to 1; Table 1).

**Table 1:** Baseline characteristics of participants

Variable^a^	HIV-uninfected and eligible for PrEP (*n=*30)
Age (years)	34 (28–38)
Gender	
Female	7 (23%)
Male	23 (77%)
Married	29 (97%)
Number of living children	3 (0–5)
Number of living children with study partner	0 (0–2)
Ethnicity	
Luo	26 (87%)
Luhya	3 (10%)
Kalenjin	1 (3.3%)
Years of schooling completed	8 (7–12)
Monthly income for participant	29 USD^b^ (5–78)
Number of years cohabitating with study partner	2 (0.42–7)
Number of months HIV serodiscordant status known	1 (1–1, range 1–72)
Number of months since first positive HIV test for study partner	9.5 (6.4–12.8)
Initiated PrEP during study	19 (63%)
Study partner on ART	21 (70%)

a *N* (%) for categorical variables; Median (IQR) for continuous variables;

b conversion rate of 1KSh=0.0098 USD used.

PrEP, pre-exposure prophylaxis.

All the study participants were aware that PrEP helps prevent HIV transmission. However, they noted that most members in their communities had no awareness of PrEP and also had a general disbelief about the existence of serodiscordant couples. A minority of participants confused PrEP with post-exposure prophylaxis, describing it as being used after car accidents or in cases of sexual assault.

Several themes emerged from the interviews with regard to 1) factors facilitating initiation of PrEP and 2) barriers, either experienced or anticipated, in initiating and adhering to PrEP. In the following, we explore these major themes in more detail with the subsequent tables containing both convergent and divergent quotations.

### Factors facilitating initiation of PrEP

Participants, particularly those who had initiated PrEP, identified several facilitators of PrEP initiation, including reducing HIV transmission, reducing stress in serodiscordant relationships, use for safer reproduction and facilitating ART initiation. Participants who had declined PrEP indicated desiring greater information, and PrEP literacy-building from the providers to help facilitate their PrEP initiation (Table 2).

**Table 2:** Factors facilitating initiation of PrEP

PrEP reduces HIV transmission	“It gives one that peace of mind when he/she wants to get intimate and knows that he/she is protected. The PrEP is a more sure way of protection because the condoms sometimes burst and are not that effective.” (HIV-uninfected female, 36 years, initiated PrEP, partner on ART)
PrEP reduces stress in and preserves relationships	“When I was being counseled, I was told that this was not the end of life because we could continue staying together in our marriage without any problems. Many people are living in HIV serodiscordant marriages and we are not the first to be affected, hence I felt relieved in my heart… I like my life and given that these drugs (PrEP) could help me prevent myself from being HIV-infected, regardless of staying with my wife, motivated me to take these drugs (PrEP).” (HIV-uninfected male, 36 years, initiated PrEP, partner on ART) “The drugs established sincerity and openness in our relationship. In fact, our HIV status didn't worry me a lot, the only thing I asked was advice on how we could live positively and maintain our relationship. That is what I consider most important.” (HIV-uninfected male, 58 years, initiated PrEP, partner on ART) “[PrEP] not only protects the HIV-negative partner but also helps many marriages to thrive. In the past, many marriages involving HIV serodiscordant couples collapsed. And another thing, it allows for conception without infecting the partner.” (HIV-infected female, 35 years, on ART, partner on PrEP)
PrEP use for safer reproduction	“You may be using the condoms for protection purposes but when it comes to desire for conception, the condoms are not effective. Therefore, if you are taking PrEP you may not have any worries when having unprotected sex because you still remain protected.” (HIV-uninfected male, 44 years, initiated PrEP, partner on ART)
PrEP use can facilitate ART initiation	“They decline to take the drugs (ARVs for PrEP or ART) because they are in denial. For instance, in my case, we went testing as a couple and, you know, that my partner would have found it hard to start taking the drug (ART). Since I accepted to start taking the (PrEP) drugs, she also decided to take the (ART) drugs … We don't have any difficulty now, but we are still surprised with the HIV (serodiscordant) results. I was not sure whether she had already known her HIV status before…” (HIV-uninfected male, 31 years, initiated PrEP, partner on ART) “Since both of us are using ARVs, we have been supportive to each other. We care for each other and ensure that neither of us misses taking his/her medication.” (HIV-uninfected male, 58 years, initiated PrEP, partner on ART)
Additional information on PrEP	“I was motivated [to start PrEP] by the fact that it was printed in the newspaper sometime back, hence, I believed that it was something real and could be effective.” (HIV-uninfected male, 36 years, initiated PrEP, partner on ART) “I think proper counseling can be one of the factors [facilitating initiation of PrEP]…. For example, if people get to know that out of 60 whose partners are positive are using PrEP and only one person is infected, and, on the other side, there are 60 couples whose partners are positive and they did not use PrEP and all their partners were infected… when people get to know of such information then they can be motivated.” (HIV-infected male, 41 years, initiated ART, partner declined PrEP) “I have never given it much thought since I trust what I was told by the care providers. I was told the importance of the pills and I do not have doubts about what I was told.” (HIV-uninfected male, 36 years, initiated PrEP, partner declined ART)

PrEP, pre-exposure prophylaxis; ART, antiretroviral therapy.

#### PrEP reduces HIV transmission

The most common reason identified as a facilitator of PrEP initiation was that PrEP offers protection from HIV acquisition. This was highlighted in the context of unprotected sex or ineffective condom use and was cited equally by those who accepted PrEP and those who declined PrEP.

#### PrEP reduces stress in and preserves relationships

Participants also often identified relationship-related factors as important reasons to initiate PrEP. For many participants, it was a way to reduce stress within the relationship, for both the infected and uninfected partners. Others felt that PrEP allowed them to preserve their marriages, mainly for those who chose to use PrEP; this was largely the case for participants who viewed HIV prevention as the responsibility of the uninfected partner or as a joint responsibility of both partners.

#### PrEP use for safer reproduction

A minority of the participants were aware that PrEP could be used by serodiscordant couples as a means of preventing HIV transmission while trying to conceive.

#### PrEP use can facilitate ART initiation

Many participants reported that using PrEP by the HIV-uninfected partner facilitated ART initiation by the HIV-infected partner. These couples found mutual support for adherence when they were both taking antiretrovirals (ARVs), which, in turn, fostered an improved sense of caring and openness in the relationship. On the other hand, some participants felt that being in denial of their serodiscordant status, particularly when they first learned of their status, can also impede PrEP or ART initiation. Some participants, who had started PrEP, explained that other couples may need more time to accept their serodiscordant status before starting treatment.

#### Additional information on PrEP

Factors outside the relationship, such as having additional information or support for PrEP, also appeared to facilitate PrEP use. At least one individual cited learning about PrEP initially from outside of the study setting, which offered greater security in its effectiveness and motivated the person to initiate PrEP. Some individuals who had not yet initiated PrEP reported needing more information and PrEP literacy-building to help them decide to start PrEP; many individuals specifically identified that one of their preferred sources of such information would be healthcare providers.

### Barriers to PrEP initiation and adherence

Both groups of participants who initiated or declined PrEP identified several barriers to PrEP initiation and adherence. Major themes included side effects; relative effectiveness of PrEP, especially in comparison to condoms; stigma and disclosure; logistics surrounding PrEP use; and associating PrEP with promiscuity or commercial sex (Table 3).

**Table 3:** Barriers to initiating and adhering to PrEP

Side effects of PrEP		“Some people say that when you start taking the drugs your body starts itching and you develop rashes. You also tend to have constant fevers. In fact, they are so many [side effects] and everyone has different reactions to the drugs.” (HIV-uninfected male, 43 years, declined PrEP, partner declined ART)
		“I can say it was the burden of taking many pills. I was on some drugs for a previous health condition I had and didn't want to combine these two medications.” (HIV-uninfected male, 36 years, declined PrEP, partner on ART)
Relative effectiveness of PrEP alongside other prevention tools		“I also did not see the sense in using both condoms and PrEP because I had always used the condoms alone yet I have never been infected. If it were that the pills are very effective and does not require me to use the condoms as well, I would have accepted. Using both methods is a challenge for me.” (HIV-uninfected female, 45 years, declined PrEP, partner on ART)
Stigma and disclosure issues	General disclosure	“Whenever a patient goes to certain sections (of a health facility) like this (one), they will have certain perceptions about you irrespective of your status. They know exactly where certain drugs are provided and the department certain types of ailments are treated or managed.” (HIV-uninfected male, 41 years, declined PrEP, partner declined ART)
		“Of course if people get to know that I am using PrEP, then it follows that they get to know my wife's status. People will talk ill about me publicly. They will say that I have been given drugs to protect myself from HIV because my wife is HIV positive.” (HIV-uninfected male, 24 years, initiated PrEP, partner declined ART)
	Disclosure of PrEP use in a relationship	“I had accepted that I should always be tested and if found to be HIV infected, then I would simply start to take my medication without any problem. But now that I am not sick, I can't take the drugs because my other two wives will ask me why I am taking such drugs yet they are not given (them). It will be as if I am lying to them that I am not sick and I can't explain anything to them.” (HIV-uninfected male, 43 years, declined PrEP, partner declined ART)
		“This is my first wife and we first got tested together and it came out that she was HIV-positive but I was not. I was therefore given PrEP but when my second wife saw these drugs she was totally convinced that I was also positive and she started telling other people that both my first wife and I were positive. This led to separation and even now as we speak she is not at home… The fact that I separated from my second wife because she was suspecting that I was HIV-positive, and she went around spreading rumors that I was infected as I was on PrEP, I consider this the worst experience.” (HIV-uninfected male, 37 years, initiated PrEP, partner on ART)
		“My first reason [for declining PrEP] is that I fear taking drugs. Secondly, the other partner I am with doesn't know that I come to this facility, and in case I start taking this drug (PrEP) every day and when I am with her, she will ask me what my problem is…” (HIV-uninfected male, 24 years, declined PrEP, partner declined ART)
		“… I have a (n extramarital) partner whose status I didn't know, so we decided to go for the test, and unfortunately, the result was positive on her side and negative on my side… There was a day that someone stabbed me with a knife late in the night and I went to the hospital for treatment but I had already been put on Truvada. So when I went back to the house, I explained to my wife that the drugs in the bottle were for treating the wounds but didn't tell her directly… She asked me why I was still taking the drugs (PrEP) even after the wound had healed, this raised her curiosity and she wanted to know what was happening… She knows the normal drugs like septrine, amoxyl, and flagyl, so she realized that these other ones (PrEP) were different.” (HIV-uninfected male, 33 years, stopped PrEP early, partner on ART)
		“If I go to take drugs (ARVs), then the other person will know and I don't want him to know. OK, just like family planning, there are those who are aware of pills, but because they are taken every day and every day they will be with their husband, they try looking for a method that will be secretive, such as coil or injection… But you find that ARVs have no such alternatives, you must take pills every day.” (HIV-infected female, 45 years, declined ART, partner declined PrEP)
	PrEP recommendation masking HIV diagnosis	“Some of them may also think that it is a trick played on them. They may think that they are actually HIV-positive but the doctor does not want to disclose that to them… They may be thinking that it is just a trick not knowing that this (PrEP) is real protection.” (HIV-uninfected male, 44 years, initiated PrEP, partner on ART)
	Not concerned with stigma	“What matters to me is that I personally know my status and whatever people think about me will not affect me in any way. Similarly, an individual who has accepted his/her status will not be affected by what other people say about him/her.” (HIV-uninfected male, 41 years, declined PrEP, partner declined ART)
		“Since most people do not know PrEP, they will think that the PrEP is ARVs. The drug known by most people is ARVs. At times, some people always wonder why I am always given so many ARVs… I usually do not hide my drugs because I do not have any fear.” (HIV-uninfected male, 24 years, initiated PrEP, partner on ART)
	Perception of promiscuity, commercial sex, or increasing risky behaviour	“I have a friend who is HIV-uninfected and the wife is HIV-infected…. He normally takes PrEP when going to have sex with an unknown partner at the town such that when he comes to the clinic for regular check-up he continues to test HIV negative…. He always asks me why I do not take this drug and I keep telling him that the drug is meant for those who are immoral like him. I can't take it because I am not a prostitute.” (HIV-uninfected male, 43 years, declined PrEP, partner declined ART)
Adherence and logistics of PrEP use		“One major disadvantage of PrEP is that the pills are taken on a daily basis, which is a burden to most people, especially, when you start using it. However, with time people adapt to taking the pills and it becomes a routine that one cannot forget.” (HIV-uninfected male, 24 years, initiated PrEP, partner declined ART)
		“Taking the drugs when in reality you are not sick feels like torture… The worst (disadvantage of PrEP) is taking the drug when you are not sick.” (HIV-uninfected female, 36 years, initiated PrEP, partner on ART)
		“The space I am talking about is in relation to my work place because I am a person who moves to places. For instance, I work four days in Kisumu and two days in Western… at the same time I cannot keep travelling with these drugs. So once I settle in one place then it can be easy for me to start taking the drugs.” (HIV-uninfected male, 24 years, declined PrEP, partner declined ART)
		“What I know is that when there is something beneficial to life, people can walk miles regardless of how long they take to get to the site. Therefore, nobody should claim that transport is an issue unless s/he is not serious.” (HIV-uninfected male, 36 years, initiated PrEP, partner on ART)
Experimental use of PrEP and racism		“I have heard people say that these drugs have not yet been approved. They are of the opinion that those who have already subscribed to the PrEP medication are just test objects, ‘guinea pigs’, being used to test the effectiveness of these drugs. To them these drugs are but preliminary tests done by the whites to determine the effectiveness of these drugs, using fellow humans as test objects.” (HIV-infected female, 22 years, initiated ART, partner declined PrEP)

PrEP, pre-exposure prophylaxis; ART, antiretroviral therapy; ARVs, antiretrovirals.

#### Side effects of PrEP

Side effects, whether experienced or perceived, were commonly named as potential barriers. Participants identified multiple side effects that they attributed to PrEP, including decreased libido or appetite, fatigue, stomach aches and rashes; some individuals reported the same side effects but in the opposing direction, such as increased libido and appetite. Other perceptions included needing greater amount of food in order to take the medications or abstaining from alcohol while using PrEP. In addition, at least one participant was concerned about pill burden, specifically taking PrEP after or concurrently with other medications. Of note, several of the participants who had initiated PrEP indicated that many of their concerns related to side effects resolved over time with continued PrEP use.

#### Relative effectiveness of PrEP alongside other prevention tools

Among participants who declined PrEP, several questioned using PrEP alongside other prevention tools, such as condom use or male circumcision. They viewed PrEP use as duplicating these other prevention tools, particularly condom use which was encouraged by the study staff. Furthermore, some participants felt that after being in a serodiscordant relationship for so many years or relying on condoms for so long, PrEP offered little additional benefit.

#### Stigma and disclosure issues

Taking PrEP was also associated with stigma, both a fear of being misidentified as HIV-infected and having one's partner's status disclosed. Participants in both groups felt that the pills were easily recognizable as ARVs, which are more commonly known for HIV treatment. In addition, being seen attending an HIV clinic itself might lead others to believe they were HIV-infected. A minority of participants indicated it being a challenge to conceal their PrEP use from their marital partners, implying they were using PrEP for prevention in extramarital partnerships. In addition, one HIV-infected female indicated that unlike some contraceptive methods (e.g. injectable methods), which can be used covertly, a barrier to taking ARVs is the difficulty in concealing daily pill use from a husband or sexual partner.

Another unique element of disclosure that arose regarding HIV-positive status and PrEP use is that a small number of participants who had initiated PrEP believed that others may have declined PrEP because these individuals think the providers are tricking them into initiating ART without telling them that they are HIV-infected. Nonetheless, there were some participants, including those who declined or accepted PrEP, who stated that they were not concerned if others knew they were taking HIV-related drugs or if their partner's status was known in the community.

Lastly, a small number of participants who declined PrEP associated it with promiscuity, commercial sex, or increasing risky behaviours. This association with “immoral” behaviour explicitly prevented them from initiating PrEP.

#### Adherence to and logistics of PrEP use

Especially among those participants who declined PrEP, there were many concerns about anticipated barriers to PrEP adherence. For instance, daily dosage, size and bitterness of the pill were all reported as concerns. Many participants in both groups questioned taking a daily medication when they were not ill. However, those who had accepted PrEP also offered suggestions on how to overcome these barriers, such as taking their medication at a routine time.

Participants identified various logistical barriers to PrEP initiation, such as having to carry pills with them or difficulties in getting to the clinic for refills. Travel (for work or personal reasons) was often mentioned as an obstacle to adherence, and in some cases, prevented individuals from initiating PrEP. There were specific work-related concerns, such as being able to keep up with the physical demands of their work, missing time from work to obtain refills or having the pills be identified at work. These concerns were reported by both those who accepted and those who declined PrEP, although those who accepted PrEP indicated that these logistical barriers could be overcome if people were serious about PrEP use.

#### Experimental use of PrEP and racism

One participant was concerned about the legitimacy of PrEP, questioning its use in a study setting as whites “experimenting” on Africans.

## Discussion

This is one of the first studies among heterosexual serodiscordant couples to investigate barriers to and facilitators of actual PrEP use. Some of the themes echo those identified in other populations, although we identified several additional themes. Our study identified three key factors that may facilitate the initiation of PrEP in this population. First, participants using PrEP noted reduced stress and increased trust in their HIV serodiscordant relationships. Second, participants felt that greater community-wide knowledge of PrEP may lead to increased PrEP acceptance. Third, participants identified that greater education and literacy-building by providers on PrEP use would likely increase the adoption of PrEP. We also identified three key barriers to PrEP initiation and adherence among heterosexual serodiscordant couples offered PrEP in Kenya. First, most participants who declined PrEP expressed doubts about the relative effectiveness of PrEP, given their current use and knowledge of other prevention tools. Second, perceived stigma related to PrEP use was an important barrier to PrEP initiation. Third, many struggled with overcoming perceived side effects or logistical challenges of taking daily PrEP, particularly when they themselves were not ill.

While all the participants identified the potential of PrEP to reduce HIV transmission, those who had accepted PrEP noted that one of the biggest benefits they experienced was reducing stress in their serodiscordant relationships and, often, facilitating remaining in those relationships. Others have shown how PrEP can facilitate trust or enhance intimacy in a relationship [8,12,25]. Interestingly, some participants noted that their PrEP use motivated their infected partners to initiate ART, which is a novel finding of our study. In the context of universal ART, initiating PrEP in the uninfected partner of the serodiscordant couple may be an additional tool programmes can use to initiate ART for the infected partner. These positive effects of PrEP use on the couple's relationship can be highlighted as a potential benefit of PrEP use.

Some participants who declined PrEP noted that additional information from providers could motivate them to initiate PrEP. Ware *et al*. [25] highlighted that counselling often helped those who were struggling with PrEP initiation or adherence; even in our study, many participants cited their clinician's advice as a reason for their PrEP initiation. In resource-limited settings, providers are often key sources of health information, particularly for sexual and reproductive health services [26]. Enhancing provider knowledge has a positive impact on provider and patient practices, and patient outcomes [27,28]. Therefore, training providers to counsel about the benefits and risks is key to wide-scale implementation of PrEP, particularly in the context of combination HIV prevention. Although medical or research mistrust emerged as a less common theme in our study, which was conducted within an implementation study of a PrEP delivery model, as compared with PrEP clinical trials testing product efficacy [13,15,17], local ownership of PrEP implementation should help abate any perceptions of experimentation, racism or neocolonialism.

One of the key barriers that we identified is participants’ doubt regarding the relative effectiveness of PrEP or its added benefits when already using other prevention tools. Convincing individuals of the relative effectiveness of PrEP is a challenge for implementation programmes. Programmes encourage concomitant condom use – a cornerstone of counselling messages for HIV prevention – which confuses potential PrEP users. Additional prevention tools, such as male circumcision and viral suppression of the infected partner, further complicate programme counselling on the added benefits of PrEP use. Counselling about combination HIV prevention is challenging and can leave patients and providers confused as to how to prioritize among the HIV prevention tools. In the context of universal ART in resource-limited settings, given the low likelihood of HIV transmission with ART use and plasma viral suppression [29], the use and messaging for PrEP may have to evolve to be even more nuanced, to be used as a bridge either during the initiation of ART or during periods of time when the infected partner may not be virally suppressed. As such, operational research on how best to counsel patients to select and combine appropriate HIV prevention strategies is urgently needed.

An important barrier to initiation of PrEP in our study was stigma associated with PrEP use. Some HIV-uninfected individuals fear being labelled as HIV-infected if identified as using PrEP, which is consistent with findings from other studies [8,11,12,15]. Some participants associated PrEP use with promiscuity or commercial sex, while others worried that PrEP use would encourage riskier sexual behaviour, findings replicated in other studies [8,12]. On the contrary, analysis from the Partners PrEP study suggests that PrEP use does not lead to sexual disinhibition or reduced condom use in heterosexual couples [30]. Nonetheless, as PrEP becomes implemented more widely in resource-limited settings, community-wide education and stigma-reduction campaigns will have to address such stigma directly and build inclusive messages of HIV prevention for different high-risk groups, including heterosexual serodiscordant couples.

Some participants who declined PrEP initiation struggled with disclosing their PrEP use to their marital partners due to the use of PrEP for extramarital sex or serodiscordance among multiple wives. Other studies have identified similar stigma and concerns regarding disclosure of PrEP use [8,11,12,15]. More covert prevention technologies, such as injectable PrEP, someday may reduce such a barrier.

Various concerns regarding perceived adherence, logistics, side effects and food insecurity challenges impeded PrEP initiation. We found that many struggled with daily dosing of PrEP [8], particularly when they did not perceive themselves as ill [11,15]. Other participants anticipated difficulties with adherence when travelling or at work [9–12]. Longer acting PrEP that maintains efficacy more than weeks or months may help alleviate such barriers to initiation or adherence. Many participants were concerned with a wide variety of physical side effects. In addition, a sense of food insecurity acted as a barrier to PrEP initiation. Specifically, some participants thought they required high caloric meals to take the PrEP with on a daily basis; the formulation offered in this demonstration trial, Truvada^®^, does not have any specific caloric food requirements [31]. Abstinence from heavy alcohol use, which is desired in general, is not a contraindication to PrEP use, though others have shown alcohol use can impede adherence [9,11,12]. Again, studies evaluating messaging, including provider counselling, on PrEP are needed to ensure that potential candidates receive and understand accurate information about PrEP use.

There are several strengths of this study. First, we conducted the interviews with participants who were eligible for PrEP use, many of whom had initiated it, rather than querying hypothetical PrEP use. Second, we conducted the study in a high HIV prevalence setting among heterosexual serodiscordant couples. Third, we conducted this study with a relatively large sample size, which ensured adequate saturation of themes. Nonetheless, we sampled participants from only one geographic region of Kenya which limits the generalizability of the results. Our results require validation with a larger and more varied sample of providers and patients.

## Conclusions

We identified several key facilitators of and barriers to the uptake of PrEP among heterosexual HIV serodiscordant couples offered PrEP in Kenya. Reduced stress and increased trust in their HIV serodiscordant relationships was seen as an important facilitator of PrEP acceptance, as did enhanced provider training on PrEP. Concerns over side effects of PrEP use, HIV-related stigma, and perceived adherence and logistical challenges were key barriers to the uptake of PrEP. Moreover, participants doubted the relative effectiveness of PrEP or its added benefits when already using other prevention tools. Further research focused on how best to provide counselling on combination HIV prevention tools in the context of PrEP use is a crucial next step to delivering PrEP in Kenya and other high HIV prevalence areas in sub-Saharan Africa. Leveraging the facilitators and overcoming barriers to PrEP uptake may enhance the successful rollout of PrEP in this region, thereby reducing sexual transmission of HIV.
